# Influences of cognitive load on center of pressure trajectory of young male adults with excess weight during gait initiation

**DOI:** 10.3389/fbioe.2023.1297068

**Published:** 2024-01-05

**Authors:** Lingyu Kong, Zhiqi Zhang, Jiawei Bao, Xinrui Zhu, Yong Tan, Xihao Xia, Qiuxia Zhang, Yuefeng Hao

**Affiliations:** ^1^ School of Physical Education, Soochow University, Suzhou, China; ^2^ School of Mathematical Sciences, Soochow University, Suzhou, China; ^3^ Rehabilitation Medicine Department, Xuzhou Rehabilitation Hospital, Xuzhou, China; ^4^ Wuxi 9th People’s Hospital Affiliated to Soochow University, Wuxi, China; ^5^ Orthopedics and Sports Medicine Center, The Affiliated Suzhou Hospital of Nanjing Medical University, Suzhou, China; ^6^ Gusu School, Nanjing Medical University, Suzhou, China

**Keywords:** cognition, gait initiation, overweight, obesity, postural control

## Abstract

**Introduction:** Falls and fall-related injuries in young male adults with excess weight are closely related to an increased cognitive load. Previous research mainly focuses on analyzing the postural control status of these populations performing cognitive tasks while stabilized walking progress but overlooked a specific period of walking known as gait initiation (GI). It is yet unknown the influences of cognitive load on this population’s postural control status during GI.

**Objective:** This study aimed to determine the influences of cognitive load on the center of pressure (CoP) trajectory of young male adults with excess weight during GI.

**Design:** A controlled laboratory study.

**Methods:** Thirty-six male undergraduate students were recruited and divided into normal-weight, overweight, and obese groups based on their body mass index (BMI). Participants’ CoP parameters during GI under single and dual-task conditions were collected by two force platforms. A mixed ANOVA was utilized to detect significant differences.

**Results:** Compared with the normal-weight group, the obese group showed significant changes in the duration and CoP parameters during sub-phases of GI, mainly reflecting prolonged duration, increased CoP path length, higher mediolateral CoP displacement amplitude, and decreased velocity of anteroposterior CoP displacement. During GI with 1-back task, significantly increased mediolateral CoP displacement amplitude occurred in the obese group. During GI with 2-back task, the obese group had increased CoP path length, higher mediolateral CoP displacement amplitude, as well as a decreased velocity of CoP displacement.

**Conclusion:** Based on the changes in CoP parameters during GI with cognitive tasks, young male adults with excess weight, mainly obese ones, have compromised postural stability. During GI with a difficult cognitive task, obese young male adults are more susceptible to deterioration in their lateral postural balance. These findings indicate that the increased cognitive load could exacerbate obese young male adults’ postural control difficulty during GI under dual-task conditions, putting them at a higher risk of experiencing incidents of falls. Based on these findings, we offer suggestions for therapists to intervene with these young male adults to ensure their safety of GI.

## 1 Introduction

At present, over 1.9 billion and more than 600 million individuals have excess weight and can be classified as overweight or obese, respectively (Barone et al., 2020). Aged 18–35 years is an influential period for excessive weight gain and unhealthy weight-related behaviors (Lytle et al., 2017). Excess weight can decrease the capacity of young adults to properly use proprioceptive information for postural control, making them have almost twice the fall risks as their normal-weight counterparts (Fjeldstad et al., 2008). In the population of young adults, males are more susceptible to falls and injuries for their slip-induced fall risks notably increased along the transversal direction under certain conditions (Wu et al., 2012). Based on these reasons, it is imperative to assess the motor performance of young male adults with excess weight to evaluate their safety and provide a theoretical basis for preventing them from experiencing dangerous events.

Gait initiation (GI), the transient period between standing posture and steady-state walking, involves the correct preparation and execution of a sequence of movements. Individuals not only need to shift their weight and transition the base of support voluntarily but also need to generate an appropriate propulsion force to reach the required gait speed and control the disequilibrium led by the walking progression (Hass et al., 2004; Colné et al., 2008). Analyzing biomechanical performance during GI in young male adults with excess weight, including the calculation of center of pressure (CoP) parameters (e.g., CoP displacement amplitude and velocity of CoP displacement), can provide deep insights into their postural control status, having important implications regarding their fall prevention (Cau et al., 2014; Hirjaková et al., 2018). However, currently, there is a lack of studies comparing the differences in CoP parameters between normal-weight and excess-weight ones during GI, which hinders researchers from gaining a deeper understanding of postural control status among these populations.

Maintaining a stable and sustained posture requires correct neuromuscular control, which relies on sufficient cognitive resources. Dual-task conditions are common in daily life, especially those involving the simultaneous performance of both cognitive and motor tasks (Brauer et al., 2001), in which cognitive tasks will compete with motor tasks for cognitive resources. This competition leads to uneven resource allocation between the two tasks, resulting in a decline in performance or quality in one or both tasks (Yogev-Seligmann et al., 2008). Even though walking is considered a motor activity that does not require a great degree of conscious thought, individuals may still be unstable and at risk of falling if they have difficulty reasonably dividing their attention into motor and cognitive tasks during walking under dual-task conditions (Fraser et al., 2017). Excessive body weight increase has been associated with a higher risk for impaired cognitive function (Volkow et al., 2009). Young male adults with excess weight have neurocognitive deficits, like impairments in the efficiency of central processing (Tsai et al., 2019), which might make them more dangerous while walking and dealing with cognitive tasks simultaneously. Numerous studies have explored the performance of individuals with excess weight during walking under dual-task conditions (Wu et al., 2016; Shaik et al., 2022). But, few studies evaluated the CoP parameters of young male adults during GI under dual-task conditions, making the underlying postural control mechanisms in young male adults with excess weight during GI under dual-task conditions remain unknown.

Cognitive load is a vital influence contributor to behaviors (Byrd-Bredbenner et al., 2016). Once cognitive load increases, the difficulty of maintaining postural stability will become more apparent, potentially impairing an individual’s ability to actively control their balance (Small et al., 2021). In previous research, influences of cognitive overload were primarily observed in the impairment of gait control, as evidenced by alterations in the gait parameters (Xu et al., 2023). Considering that excess weight is usually accompanied by cognitive function inhibition compared with normal-weight peers (Moss et al., 2023; Wernberg et al., 2023), this means that the increased cognitive load may specifically influence the postural control of overweight or obese young male adults, putting them at a higher risk of falling. Some researchers have suggested that future research should thoroughly analyze secondary cognitive tasks with different difficulty levels to better understand the GI performance of individuals with excess weight under dual-task conditions (Qu et al., 2021). However, this aspect has not been explored yet.

In summary, the purpose of the current study was to further investigate the differences in CoP parameters among young male adults with normal weight and excess weight during GI with different difficult levels of cognitive tasks, determining the influences of cognitive load on CoP parameters of young male adults with excess weight during GI. We made three assumptions.

1) There are differences in CoP parameters during GI between normal-weight and young male adults with excess weight.
2) During GI with cognitive tasks, young male adults with normal weight and those with excess weight had different CoP parameters.
3) During GI with a higher cognitive load, obese young male adults may have different CoP parameters compared with those who are normal weight or even overweight.

## 2 Materials and methods

### 2.1 Participants

In this study, thirty-six male undergraduate students were recruited from Soochow University. The study applied the following inclusion criteria: i) individuals aged between 18 and 35 years (Lytle et al., 2017), ii) individuals with normal or corrected-to-normal vision, iii) no history of neurological disorders, and iv) no history of musculoskeletal disorders, as well as no self-reported severe physical diseases that could impede locomotion. After explaining the experimental protocol, all participants signed written informed consent before the experiment. They were evenly classified into the normal-weight group, overweight group, and obese group, according to the classification criteria for overweight and obesity in China [i.e., normal-weight: 18.5≤ body mass index (BMI) ≤23.9 kg/m^2^; overweight: 24.0≤ BMI ≤27.9 kg/m^2^; obesity: BMI ≥28.0 kg/m^2^] (National Health and Family Planning Commission of the People’s Republic of China, 2013). The researchers asked participants to walk three times consecutively from a standing position in order to determine their preferred swung leg during GI. The participants’ data is presented in Table 1. This study received ethical approval from the Soochow University Human Research Institutional Review Board.

**TABLE 1:** General characteristics of participants.

Characteristics	Body weight			*F*-value	*p*-value
	Normal-weight (*n* = 12)	Overweight (*n* = 12)	Obese (*n* = 12)		
Age (years)	22.8 ± 2.7	22.9 ± 3.0	22.4 ± 2.5	0.113	0.894
Height (m)	1.72 ± 0.06	1.71 ± 0.04	1.73 ± 0.07	0.514	0.603
Weight (kg)	62.1 ± 7.7	74.5 ± 5.7	93.2 ± 8.1	56.246	**<0.001** ^a^
BMI (kg/m^2^)	20.9 ± 1.5	25.6 ± 1.5	31.1 ± 1.4	152.577	**<0.001** ^a^
Preferred swing leg during GI					
Left	3	7	5	—	
Right	9	5	7		

^a^ *p*-values < 0.017 for normal-weight group vs. overweight group vs. obese group.

The meaning of the bold values is that significant statistical differences exist.

### 2.2 Test equipment

A screen (U55H3, Haier, China) was set at the endpoint of a 5-m linear walkway (Simonet et al., 2022). CoP parameters during GI were collected by two force platforms (9287B, KISTLER, Switzerland) sequentially embedded in the walkway along the progression direction 1 cm apart. An 8-camera motion capture system (Vicon, Oxford, United Kingdom) was used to collect spatial data from reflective markers placed on the participants’ feet, following the scheme provided by the Conventional Gait Model 2.0 (Vicon 2020). The motion capture system and force platforms were synchronized with the sampling rates at 100 and 1,000 Hz, respectively.

### 2.3 N-back task

The N-back is a continuous performance task requiring high cognitive resources. In this study, a series of letters (from “A” to “J”) were randomly presented in a sequence using a visual procedure on the screen. Participants had to monitor these letters and decide whether each letter in a sequence was consistent with the one that appeared N steps ago. The presentation of letters was controlled by E-Prime software 2.0 (Psychology Software Tools, Sharpsburg, PA, United States). Before the presentation of each letter, a cross character appeared on the screen for 500 ms. Subsequently, the letter was displayed for 500 ms, and the participants were given 1,500 ms to respond. The N-back tasks used in this study were constructed using lists of 25 plus N letters, comprising 20 percent “yes” and 80 percent “no” responses (Wrightson et al., 2016). Additionally, the list used in each experiment was different.

Cognitive tasks consist of two difficulty levels: easy (1-back task) and difficulty (2-back task), which require low and high cognitive demand and impose different levels of cognitive load during walking-related movements, respectively (Patelaki et al., 2023). For 1-back task, if the current letter presented is consistent with the previous one, that is a “target” stimulus requiring a “yes” response; once the current letter presented is inconsistent with the previous one, that is, a “not target” stimulus, the participants have to make a “no” response (Nocera et al., 2013). Similarly, for 2-back task, participants have to compare the current letter to the one presented two steps earlier and make a “yes” or “no” response (Nocera et al., 2013). The first letter of 1-back task and the first two letters of 2-back task did not require a response.

### 2.4 Experimental procedure

Before the formal experiment, participants were given 10 min to train in the N-back task to familiarize themselves with the procedure of N-back tasks. In the formal experiment, the participants started each trial by standing barefoot on the first force platform. They maintained an upright posture with their arms at their sides, fixed their heads in a neutral position, and looked straight ahead with their eyes. The initial positioning of the feet was self-selected and then subsequently marked to ensure consistent foot placement and stance width throughout the experimental procedure. Each participant walked from the start point to the endpoint under single and dual-task conditions.

#### 2.4.1 Single-task condition

Participants stood on the force plate, as mentioned above. Once a letter with an illuminated light appeared on the screen, participants needed to initiate gait and walk along the 5-m walkway. A successful trial under the single-task condition was defined as one where the participants’ preferred swung leg during GI correctly stepped on the second force platform, with the foot not exceeding the edge of the force platform, and test equipment successfully captured trial data.

#### 2.4.2 Dual-task conditions

**FIGURE 1:**
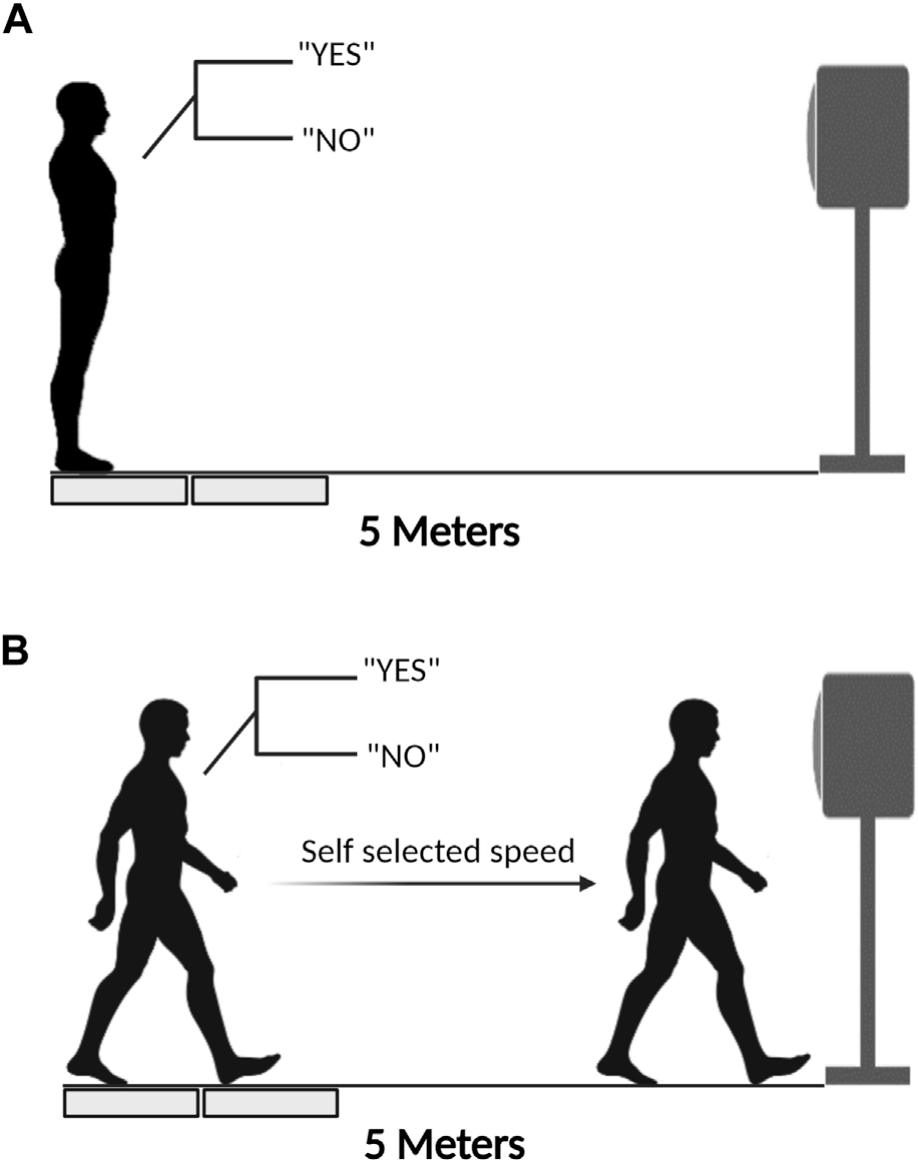
The experimental procedure during GI under dual-task conditions. **(A)**: Participants stand on the force platform and respond according to the N-back task requirements; **(B)**: Make a response according to the N-back task requirements and initiate gait simultaneously.

Participants stood on the force plate, as described above. According to the requirements of the N-back test, participants need to respond continuously as accurately and quickly as possible (Figure 1A). No feedback was given during or after each trial or block. Until the end of the list, the letter with an illuminated light would appear and cue participants to initiate gait. Participants must respond correctly, followed by the N-back test requirement, and immediately initiate gait and walk to the endpoint (Figure 1B). A 5-min gap was set between each 1- and 2-back task, allowing participants to relax. A successful trial under the dual-task condition was defined as one in which participants achieved complete accuracy in the N-back tasks (to ensure that participants really focused on performing cognitive tests) and their preferred swung leg during GI stepped on the second force platform, with their foot not exceeding the edge of the force platform, and test equipment successfully captured trial data. Those trials of participants who fail to meet the accuracy requirement of N-back tasks during GI with cognitive tasks will not be used for analysis. Finally, all participants need to conduct three successful trials under single- and dual-task conditions, respectively.

### 2.5 Dependent variables

The data obtained from successful trials were analyzed using MATLAB software (R2023a, MATLAB, Mathworks, Inc., Natick, MA, United States). Moment and force components were low-pass filtered at 10 Hz (Butterworth, fourth order), and then CoP parameters were calculated.

**FIGURE 2:**
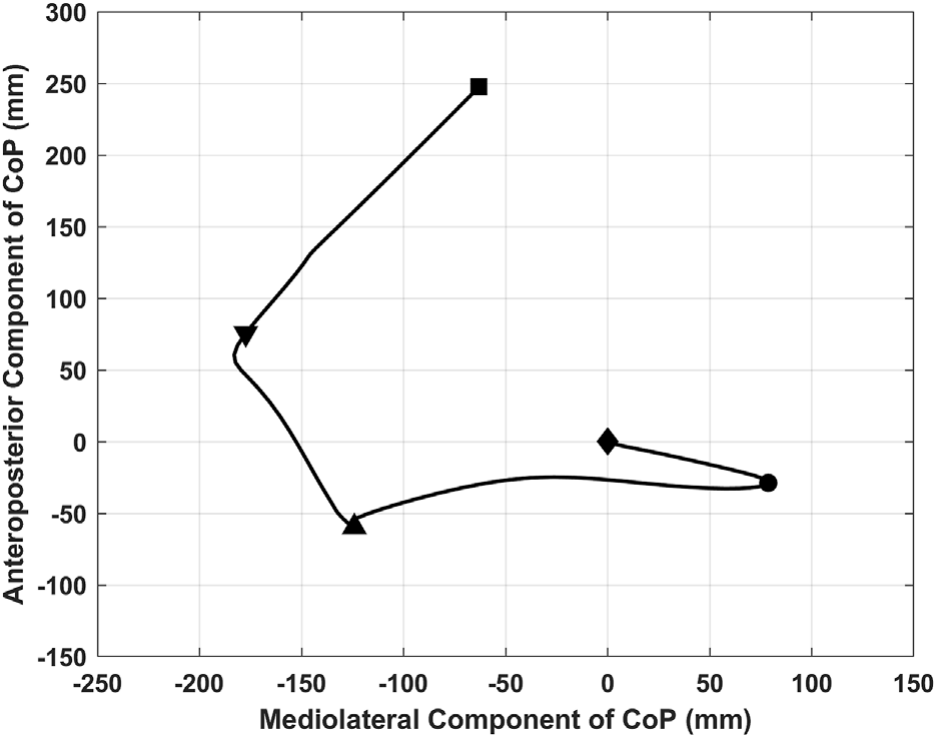
The division of GI and the meanings of landmarks. ♦: Onset of the GI period and the imbalance phase; ●: End of the imbalance phase and onset of the unloading phase; ▲: Onset of the monopedal phase and the end of the unloading phase; ▼: End of the monopedal phase and the onset of the bipedal phase; ■: End of the bipedal phase and the GI period.

According to five landmarks, the GI was divided into four sub-phases. Specifically, those landmarks were successively representing: 1) the CoP started deviation toward the swing leg; 2) the CoP completely under the rear foot of the swing leg; 3) the heel of the swing leg left off the ground; 4) the foot of the swing leg contacted ground; and 5) the toe of the stance leg left from the ground. The four sub-phases are sequentially named as imbalance, unloading, monopedal standing, and bipedal standing phases. The imbalance phase was characterized by the CoP deviation towards the swing leg, followed by a transfer to the posterior end of the swinging leg. Deviation onset was determined as the time point when mediolateral (ML) CoP displacement exceeded three standard deviations from its baseline. The baseline was calculated as the mean value of ML CoP displacement in the 1,500 ms period before the visual cue of the last letter was presented (Uemura et al., 2012; Yousefi et al., 2020). The unloading phase represented the movement of the CoP towards the initial stance foot and stopped under the initial stance foot. The monopedal standing phase started with the forward displacement of CoP, which was at the instant of transition to the single-limb stance and ended with the heel strike of the swing leg (Davidson and Wolpert, 2005). Swing leg heel contact was determined as the moment when the vertical ground reaction force measured by the second force platform exceeded 10 N (Qu et al., 2021). The bipedal standing phase started from the forward shift of CoP and continued until the toe-off of the stance limb (Davidson and Wolpert, 2005). Stance leg toe-off was the moment when the toe marker increased by 10 mm in the vertical direction from static upright standing. The division of GI is shown in Figure 2.

Dependent variables for the assessment included spatial-temporal variables of sub-phases: duration, CoP length path, CoP speed, anteroposterior (AP) and ML CoP displacement amplitude, and velocity of AP and ML CoP displacement. Among them, AP and ML CoP displacement amplitude were calculated using Eqs 1, 2. In these equations, x and y represent the CoP position in the AP and ML directions, respectively.

$$\text{AP CoP displacement amplitude }\left(\text{cm}\right)=\left|y\;\text{end}-y\;\text{onset}\right|$$ (1)

$$\text{ML CoP displacement amplitude }\left(\text{cm}\right)=\left|x\;\text{end}-x\;\text{onset}\right|$$ (2)

The velocity of AP and ML CoP displacement were calculated using Eqs 3, 4. The variable n represents the number of data points, and 1,000 is the sampling frequency.

$$\text{Velocity of AP CoP displacement }\left(\frac{\text{cm}}{s}\right)=\frac{\text{AP}\;\text{CoP}\;\text{displacement}\;\text{amplitude}*1000}{\left(n_{\text{end}}-n_{\text{onset}}\right)}$$ (3)

$$\text{Velocity of ML CoP displacement }\left(\frac{\text{cm}}{s}\right)=\frac{\text{ML}\;\text{CoP}\;\text{displacement}\;\text{amplitude}*1000}{\left(n_{\text{end}}-n_{\text{onset}}\right)}$$ (4)

### 2.6 Statistical analysis

The mean value of the three successful trials under single- and dual-task conditions, respectively, was used for statistical analysis. Data analysis was performed using SPSS version 26 for Windows (SPSS Inc., Chicago, IL, United States). Continuous variables were presented as mean ± standard deviation (SD). The data distribution for each variable was assessed using the Shapiro-Wilk test. If the data distribution was non-normal, it was transformed for subsequent analysis using the Box-Cox transformation, to become normally distributed, i.e.,

$$y^{T}=\left\{\begin{array}{c} \frac{y^{\lambda }-1}{\lambda },\;\lambda \neq 0;\\ \ln y,\;\lambda =0, \end{array}\right.$$

where y is the original variable, $y^{T}$ represents the corresponding transformed variable, and λ is a parameter, which is supposed to be most efficient when maximizing the log-likelihood. Those non-normal variables that need Box-Cox transformed were marked in Tables, and their corresponding optimal λ values and confidence intervals can be found in the Supplementary Material.

A mixed ANOVA with a Greenhouse-Geisser correction was utilized to detect the between-subjects effect of group (normal-weight, overweight, and obese), the within-subjects effect of task condition (baseline, 1-back, and 2-back), and the interaction effect between group and task. Statistical significance was concluded when *p*-values < 0.05, and Eta partial square (*η* ^2^) was used to display the effect size. Post-hoc comparisons among groups and tasks were applied Bonferroni correction, and statistical significance was concluded when *p*-values < 0.017. In case a significant interaction was detected, a simple effects analysis was conducted. During the simple effects analysis, when performing the *post hoc* analysis among groups, the statistical significance was set at *p*-values < 0.0056.

## 3 Results

### 3.1 Duration

There were significant main effects of the group on the duration of the imbalance phase (*F* = 4.086, *p* = 0.026, *η* ^2^ = 0.198) and the bipedal standing phase (*F* = 7.262, *p* = 0.002, *η* ^2^ = 0.306) (Table 2). Post hoc analysis found no significant difference in the paired comparison between any two groups regarding the duration of the imbalance phase (*p* > 0.017); The obese group (0.24 ± 0.05 s) showed a more prolonged duration of the bipedal standing phase than the normal-weight group (0.19 ± 0.04 s) (*p* = 0.002).

**TABLE 2:** Results of the duration of sub-phases of GI (unit: s).

Variables	Task	Body weight			Main effect *p*-value		Interaction effect *p*-value
		Normal-weight (n = 12)	Overweight (n = 12)	Obese (n = 12)			
					Group	Task	Group × task
Imbalance phase^a^	Single	0.21 ± 0.07	0.23 ± 0.04	0.24 ± 0.06	**0.026**	**<0.001** ^c,d^	0.814
	1-back	0.23 ± 0.06	0.28 ± 0.07	0.28 ± 0.07			
	2-back	0.25 ± 0.06	0.31 ± 0.07	0.31 ± 0.07			
Unloading phase^a^	Single	0.36 ± 0.21	0.32 ± 0.14	0.28 ± 0.07	0.778	0.958	0.791
	1-back	0.30 ± 0.10	0.26 ± 0.05	0.30 ± 0.05			
	2-back	0.30 ± 0.07	0.28 ± 0.06	0.29 ± 0.06			
Monopedal standing phase	Single	0.30 ± 0.09	0.31 ± 0.05	0.31 ± 0.05	0.218	0.568	0.443
	1-back	0.27 ± 0.09	0.31 ± 0.04	0.29 ± 0.08			
	2-back	0.26 ± 0.07	0.32 ± 0.08	0.31 ± 0.08			
Bipedal standing phase	Single	0.18 ± 0.04	0.20 ± 0.03	0.21 ± 0.04	**0.002** ^b^	**0.002** ^d^	0.132
	1-back	0.19 ± 0.05	0.21 ± 0.02	0.24 ± 0.05			
	2-back	0.19 ± 0.03	0.21 ± 0.04	0.26 ± 0.04			

^a^ Box-Cox transformation was implemented to this variable.

^b^ *p*-values <0.017 for normal-weight group vs. obese group.

^c^ *p*-values <0.017 for single task vs. 1-back task.

^d^ *p*-values <0.017 for single task vs. 2-back task.

The meaning of the bold values is that significant statistical differences exist.

There were significant main effects of the task on the duration of the imbalance phase (*F* = 13.871, *p* < 0.001, *η* ^2^ = 0.296) and the bipedal standing phase (*F* = 7.534, *p* = 0.002, *η* ^2^ = 0.186) (Table 2). Post hoc analysis found that the duration of the imbalance phase with 1-back task (0.26 ± 0.07 s) and 2-back task (0.29 ± 0.07 s) was more prolonged than that with single task (0.23 ± 0.06 s) (*p* = 0.004; *p* < 0.001); The duration of the bipedal standing phase with 2-back task (0.22 ± 0.05 s) was more prolonged than that with single task (0.20 ± 0.04 s) (*p* = 0.009).

### 3.2 CoP path length and CoP speed

There were significant main effects of the group on the CoP path length during the imbalance phase (*F* = 5.128, *p* = 0.012, *η* ^2^ = 0.237), the unloading phase (*F* = 3.949, *p* = 0.029, *η* ^2^ = 0.193), and the bipedal standing phase (*F* = 8.337, *p* = 0.001, *η* ^2^ = 0.336) (Table 3). Post hoc analysis found that the obese group (8.34 ± 2.60 cm) showed more increased CoP path length during the imbalance phase than the normal-weight group (5.81 ± 2.41 cm) (*p* = 0.010); No significant differences were found in the paired comparison between any two groups regarding the CoP path length during the unloading phase (*p* > 0.017); The obese group (42.27 ± 5.16 cm) showed more increased CoP path length during the bipedal standing phase than the normal-weight group (35.62 ± 6.34 cm) (*p* = 0.001).

**TABLE 3:** Results of CoP path length and CoP speed (unit: cm and cm/s).

Variables	Task	Body weight			Main effect *p*-value		Interaction effect *p*-value
		Normal-weight (n = 12)	Overweight (n = 12)	Obese (n = 12)			
					Group	Task	Group × task
CoP path length							
Imbalance phase	Single	5.72 ± 2.68	6.96 ± 3.13	7.42 ± 3.28	**0.012** ^b^	**0.049**	0.664
	1-back	5.29 ± 2.37	7.41 ± 1.91	8.43 ± 2.59			
	2-back	6.41 ± 2.23	7.75 ± 2.04	9.18 ± 1.53			
Unloading phase^a^	Single	17.24 ± 5.51	18.41 ± 4.47	18.18 ± 2.76	**0.029**	0.343	**0.039**
	1-back	15.45 ± 3.98	16.79 ± 3.84	19.63 ± 2.97			
	2-back	15.90 ± 3.28	17.24 ± 4.40	21.18 ± 2.69			
Monopedal standing phase^a^	Single	11.82 ± 3.74	12.79 ± 2.76	13.29 ± 4.57	0.972	0.670	0.397
	1-back	12.91 ± 3.46	12.28 ± 1.97	11.74 ± 3.44			
	2-back	12.06 ± 3.43	12.58 ± 3.34	12.17 ± 3.54			
Bipedal standing phase^a^	Single	34.87 ± 7.44	41.79 ± 2.84	43.95 ± 6.12	**0.001** ^b^	0.204	0.055
	1-back	35.36 ± 6.62	40.08 ± 4.57	43.17 ± 4.21			
	2-back	36.64 ± 5.19	40.48 ± 4.44	39.68 ± 4.28			
CoP speed							
Imbalance phase^a^	Single	30.98 ± 18.16	31.12 ± 13.98	32.86 ± 16.32	0.354	0.427	0.611
	1-back	23.61 ± 10.52	27.33 ± 7.17	33.36 ± 16.10			
	2-back	26.11 ± 7.08	25.37 ± 7.45	31.28 ± 9.77			
Unloading phase^a^	Single	64.21 ± 41.20	65.85 ± 34.75	68.57 ± 19.73	0.099	0.731	0.756
	1-back	55.39 ± 16.83	64.58 ± 15.91	68.28 ± 16.46			
	2-back	54.64 ± 14.07	63.63 ± 17.04	77.02 ± 18.45			
Monopedal standing phase	Single	42.59 ± 17.12	43.00 ± 12.19	44.03 ± 14.84	0.387	0.881	0.211
	1-back	50.81 ± 14.30	40.43 ± 8.37	40.51 ± 12.60			
	2-back	47.01 ± 5.61	40.96 ± 15.62	40.50 ± 12.80			
Bipedal standing phase^a^	Single	194.82 ± 34.06	213.09 ± 31.05	218.79 ± 53.76	0.350	**0.001** ^c^	**0.010**
	1-back	191.90 ± 45.80	195.42 ± 37.78	183.54 ± 44.05			
	2-back	195.26 ± 38.17	196.38 ± 38.09	158.37 ± 31.38			

^a^ Box-Cox transformation was implemented to this variable.

^b^ *p*-values <0.017 for normal-weight group vs. obese group.

^c^ *p*-values <0.017 for single task vs. 2-back task.

The meaning of the bold values is that significant statistical differences exist.

There were significant main effects of the task on the CoP path length during the imbalance phase (*F* = 3.157, *p* = 0.049, *η* ^2^ = 0.087), and the CoP speed during the bipedal standing phase (*F* = 7.943, *p* = 0.001, *η* ^2^ = 0.194) (Table 3). Post hoc analysis found that no significant differences were found in the paired comparison between any two tasks regarding the CoP path length during the imbalance phase (*p* > 0.017); The CoP speed during the bipedal standing phase with 2-back task (183.33 ± 39.29 cm/s) was more decreased than that with single task (208.90 ± 41.03 cm/s) (*p* = 0.003).

**FIGURE 3:**
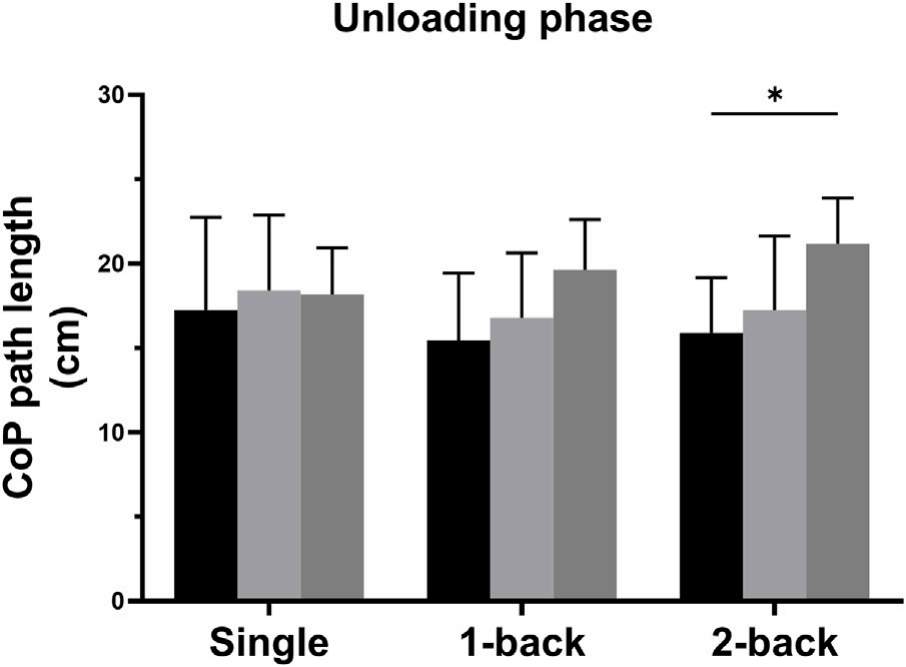
Simple analysis results in CoP path length during the unloading phase. * *p*-values < 0.0056.

There were also significant interaction effects between task and group on the CoP length path during the unloading phase (*F* = 2.685, *p* = 0.039, *η* ^2^ = 0.140) and the CoP speed during the bipedal standing phase (*F* = 3.618, *p* = 0.010, *η* ^2^ = 0.180) (Table 3). Simple effects analysis displayed that the CoP path length during the unloading phase with single and 1-back task had no significant differences among the three groups; The obese group (21.18 ± 2.69 cm) showed more increased CoP path length during the unloading phase with 2-back task than the normal-weight group (15.90 ± 3.28 cm) (*p* = 0.002) (Figure 3). The CoP speed during the bipedal standing phase with single, 1- and 2-back tasks had no significant differences between any two groups (*p* > 0.0056).

### 3.3 CoP displacement amplitude

There were significant main effects of the group on the ML CoP displacement amplitude during the imbalance phase (*F* = 6.258, *p* = 0.005, *η* ^2^ = 0.275), the unloading phase (*F* = 5.346, *p* = 0.010, *η* ^2^ = 0.245), and the bipedal standing phase (*F* = 17.631, *p* < 0.001, *η* ^2^ = 0.517) (Table 4). Post hoc analysis found that the obese group (6.63 ± 2.19 cm) showed higher ML CoP displacement amplitude during the imbalance phase than the normal-weight group (4.13 ± 2.12 cm) (*p* = 0.004); The obese group (18.73 ± 2.96 cm) showed higher ML CoP displacement amplitude during the unloading phase than the normal-weight group (14.21 ± 4.29 cm) (*p* = 0.008); The obese group (26.60 ± 4.77 cm) showed higher ML CoP displacement amplitude during the bipedal standing phase than the normal-weight group (17.06 ± 3.73 cm) (*p* < 0.001) and the overweight group (21.34 ± 4.16 cm) (*p* = 0.008).

**TABLE 4:** Results of CoP displacement amplitude (unit: cm).

Variables	Task	Body weight			Main effect *p*-value		Interaction effect *p*-value
		Normal-weight (n = 12)	Overweight (n = 12)	Obese (n = 12)			
					Group	Task	Group × task
Imbalance phase							
AP	Single	2.86 ± 1.83	3.46 ± 1.49	3.83 ± 2.36	0.171	0.208	0.906
	1-back	3.31 ± 1.55	4.02 ± 1.12	4.00 ± 1.56			
	2-back	3.21 ± 1.52	4.27 ± 1.17	4.21 ± 1.50			
ML	Single	4.51 ± 2.34	5.65 ± 2.74	5.54 ± 2.21	**0.005** ^b^	0.187	**0.039**
	1-back	3.64 ± 2.12	5.86 ± 1.55	6.96 ± 2.17			
	2-back	4.26 ± 1.97	5.88 ± 1.68	7.40 ± 1.88			
Unloading phase							
AP^a^	Single	2.72 ± 1.36	2.87 ± 1.63	1.80 ± 1.27	0.114	0.248	0.133
	1-back	2.96 ± 1.66	1.69 ± 1.03	1.29 ± 0.84			
	2-back	2.92 ± 2.40	1.72 ± 1.22	2.17 ± 1.50			
ML	Single	15.21 ± 5.45	16.49 ± 4.43	17.25 ± 2.78	**0.010** ^b^	0.311	**0.022**
	1-back	13.52 ± 3.98	15.89 ± 3.73	18.76 ± 2.88			
	2-back	13.90 ± 3.38	16.48 ± 4.43	20.18 ± 2.69			
Monopedal standing phase							
AP^a^	Single	10.70 ± 3.48	11.83 ± 2.69	11.98 ± 4.12	0.968	0.681	0.300
	1-back	11.83 ± 3.24	11.21 ± 1.94	10.61 ± 3.11			
	2-back	11.16 ± 3.43	11.59 ± 3.36	10.81 ± 3.12			
ML^a^	Single	2.45 ± 1.72	2.56 ± 1.32	3.26 ± 1.43	0.186	0.346	0.729
	1-back	2.03 ± 1.58	2.66 ± 1.43	2.85 ± 1.71			
	2-back	1.99 ± 1.41	2.19 ± 1.23	3.06 ± 1.30			
Bipedal standing phase							
AP^a^	Single	30.23 ± 6.39	34.63 ± 4.44	34.23 ± 5.13	0.245	**0.021** ^d^	0.059
	1-back	30.17 ± 5.95	33.19 ± 4.61	32.85 ± 2.33			
	2-back	31.20 ± 5.32	32.19 ± 4.87	29.58 ± 2.94			
ML	Single	16.34 ± 4.82	21.71 ± 4.19	26.39 ± 5.36	**<0.001** ^b, c^	0.345	0.673
	1-back	17.15 ± 3.76	20.65 ± 3.44	26.46 ± 5.38			
	2-back	17.70 ± 2.41	21.67 ± 4.99	26.97 ± 3.80			

^a^ Box-Cox transformation was implemented to this variable.

^b^ *p*-values <0.017 for normal-weight group vs. obese group.

^c^ *p*-values <0.017 for overweight group vs. obese group.

^d^ *p*-values <0.017 for single task vs. 2-back task.

The meaning of the bold values is that significant statistical differences exist.

There was significant main effects of the task on the AP CoP displacement amplitude during the bipedal standing phase (*F* = 4.092, *p* = 0.021, *η* ^2^ = 0.110) (Table 4). Post hoc analysis found that the AP CoP displacement amplitude during the bipedal standing phase with 2-back task (22.11 ± 5.38 cm) was lower than that with single task (21.48 ± 6.26 cm) (*p* = 0.005).

**FIGURE 4:**
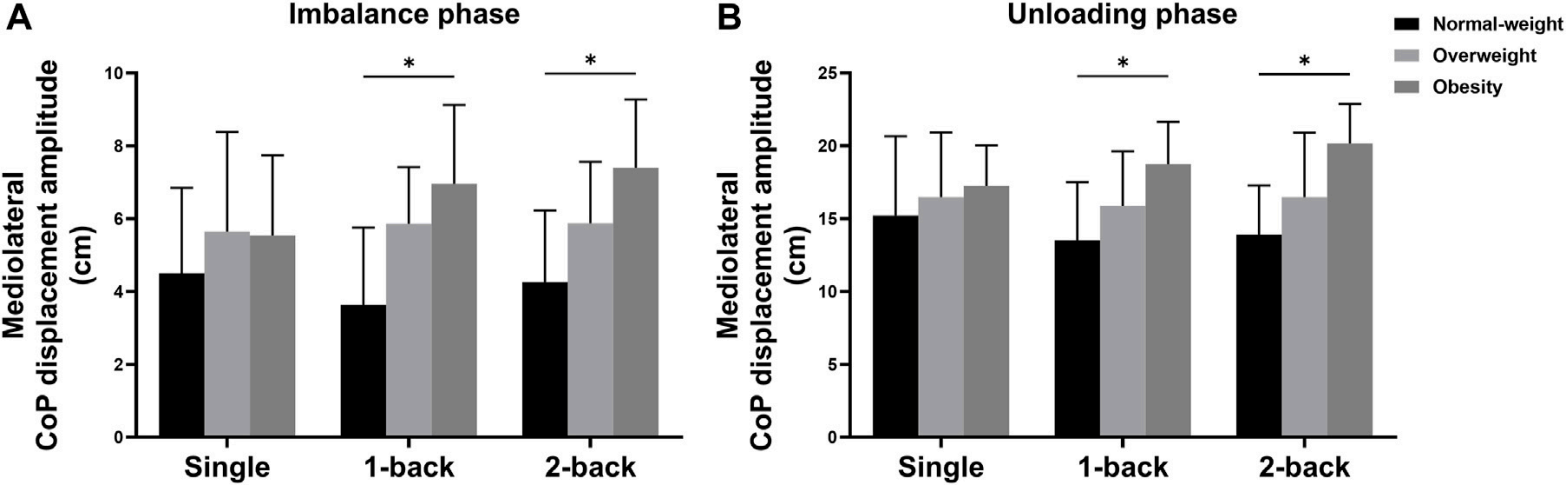
Simple analysis results in **(A)** ML CoP displacement amplitude during the imbalance phase and **(B)** ML CoP displacement amplitude during the unloading phase. * *p*-values < 0.0056.

There were also significant interaction effects between task and group on the ML CoP displacement amplitude during the imbalance phase (*F* = 2.690, *p* = 0.039, *η* ^2^ = 0.140) and the unloading phase (*F* = 3.060, *p* = 0.022, *η* ^2^ = 0.156). Simple effects analysis displayed that the ML CoP displacement amplitude during the imbalance phase with single task had no statistical differences among the three groups. The obese group (6.96 ± 2.17 cm) showed higher ML CoP displacement amplitude during the imbalance phase with 1-back task than the normal-weight group (3.64 ± 2.12 cm) (*p* = 0.001); The obese group (7.40 ± 1.88 cm) showed higher ML CoP displacement amplitude during the imbalance phase with 2-back task than the normal-weight group (4.26 ± 1.97 cm) (*p* = 0.001) (Figure 4A). Additionally, the ML CoP displacement amplitude during the unloading phase with single task had no significant differences among the three groups. The obese group (18.76 ± 2.88 cm) showed higher ML CoP displacement amplitude during the unloading phase with 1-back task than the normal-weight group (13.52 ± 3.98 cm) (*p* = 0.003); The obese group (20.18 ± 2.69 cm) showed higher ML CoP displacement amplitude during the unloading phase with 2-back task than the normal-weight group (13.90 ± 3.38 cm) (*p* < 0.001) (Figure 4B).

### 3.4 Velocity of CoP displacement

There was a significant main effects of the group on the velocity of AP CoP displacement during the bipedal standing phase (*F* = 3.716, *p* = 0.035, *η* ^2^ = 0.184) (Table 5). Post hoc analysis found no significant difference in the paired comparison between any two groups regarding the velocity of AP CoP displacement during the bipedal standing phase (*p* > 0.017).

**TABLE 5:** Results of velocity of CoP displacement (unit: cm/s).

Variables	Task	Body weight			Main effect *p*-value		Interaction effect *p*-value
		Normal-weight (n = 12)	Overweight (n = 12)	Obese (n = 12)			
					Group	Task	Group × task
Imbalance phase							
AP^a^	Single	15.74 ± 11.86	15.61 ± 7.49	17.17 ± 11.61	0.899	0.573	0.993
	1-back	14.79 ± 7.39	14.99 ± 5.03	15.64 ± 7.87			
	2-back	13.55 ± 6.34	14.22 ± 5.42	14.88 ± 7.91			
ML^a^	Single	24.50 ± 14.93	25.29 ± 12.08	24.97 ± 12.51	0.172	0.135	0.146
	1-back	16.34 ± 9.20	21.60 ± 5.62	27.66 ± 13.63			
	2-back	17.74 ± 7.47	19.16 ± 5.63	25.13 ± 8.40			
Unloading phase							
AP^a^	Single	10.47 ± 8.28	9.66 ± 7.28	6.18 ± 3.55	0.167	0.481	0.357
	1-back	9.78 ± 4.59	6.58 ± 4.44	4.61 ± 3.43			
	2-back	10.23 ± 9.48	6.47 ± 5.06	8.61 ± 8.20			
ML^a^	Single	58.62 ± 41.11	60.92 ± 35.54	65.35 ± 19.96	0.051	0.761	0.675
	1-back	49.33 ± 17.64	61.14 ± 15.60	65.34 ± 16.42			
	2-back	47.96 ± 14.57	60.84 ± 16.96	73.38 ± 17.83			
Monopedal standing phase							
AP	Single	38.42 ± 15.38	39.70 ± 11.49	39.74 ± 13.58	0.360	0.866	0.145
	1-back	46.68 ± 13.65	37.05 ± 8.98	36.44 ± 11.34			
	2-back	43.37 ± 6.32	37.47 ± 14.44	36.13 ± 12.14			
ML^a^	Single	9.71 ± 7.61	8.51 ± 4.90	10.94 ± 5.12	0.468	0.465	0.896
	1-back	7.98 ± 6.04	8.56 ± 4.30	10.04 ± 6.87			
	2-back	7.95 ± 5.78	7.84 ± 5.49	10.19 ± 4.43			
Bipedal standing phase							
AP^a^	Single	169.37 ± 33.22	177.01 ± 32.92	171.06 ± 46.33	**0.035**	**<0.001** ^b, c^	**0.007**
	1-back	163.37 ± 40.92	161.22 ± 29.28	139.04 ± 28.52			
	2-back	165.88 ± 35.35	155.90 ± 34.47	118.03 ± 22.00			
ML^a^	Single	91.05 ± 20.89	110.33 ± 23.74	130.75 ± 34.43	0.077	0.111	0.189
	1-back	94.74 ± 27.52	101.17 ± 25.68	113.70 ± 39.62			
	2-back	95.62 ± 22.97	105.12 ± 28.82	108.11 ± 25.96			

^a^ Box-Cox transformation was implemented to this variable.

^b^ *p*-values <0.017 for single task vs. 1-back task.

^c^ *p*-values <0.017 for single task vs. 2-back task.

The meaning of the bold values is that significant statistical differences exist.

There was a significant main effects of the task on the velocity of AP CoP displacement during the bipedal standing phase (*F* = 11.652, *p* < 0.001, *η* ^2^ = 0.261) (Table 5). Post hoc analysis found that the velocity of AP CoP displacement during the bipedal standing phase with 1-back task (154.54 ± 34.29 cm/s) and 2-back task (146.60 ± 36.81 cm/s) were more decreased than that with single task (172.48 ± 37.06 cm/s) (*p =* 0.012; *p <* 0.001).

**FIGURE 5:**
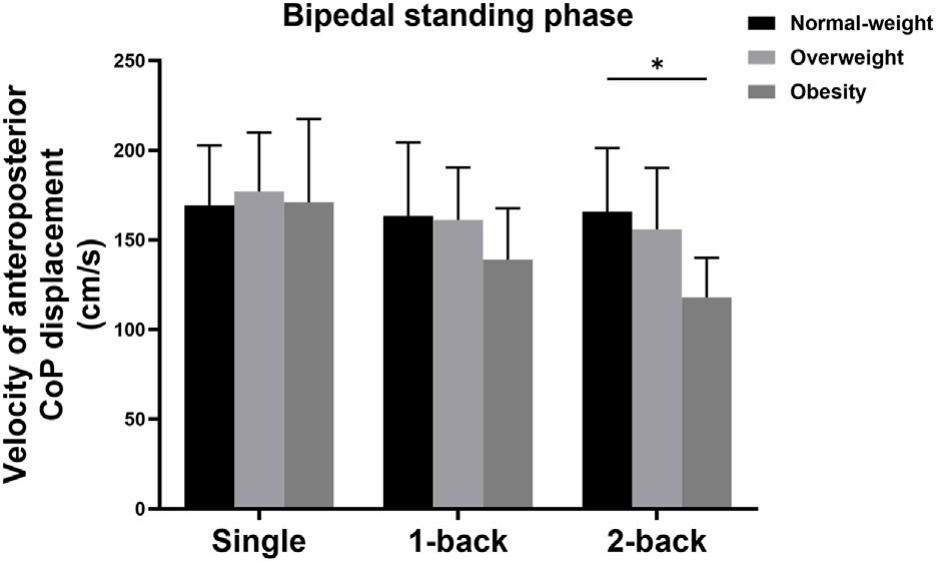
Simple analysis results in velocity of AP CoP displacement during the bipedal standing phase. * *p*-values < 0.0056.

There was also a significant interaction effect between group and task on the velocity of AP displacement during the bipedal standing phase (*F* = 3.869, *p* = 0.007, *η* ^2^ = 0.190). Simple effects analysis displayed that the velocity of AP displacement during the bipedal standing phase with single and 1-back tasks showed no statistical differences among the three groups; The obese group (118.03 ± 22.00 cm/s) showed more decreased velocity of AP displacement during the bipedal standing phase with 2-back task than the normal-weight group (165.88 ± 35.35 cm/s) (*p* = 0.001) (Figure 5).

## 4 Discussion

### 4.1 Influence of excess weight on young male adults’ CoP parameters

Consistent with our assumption 1, differences in CoP parameters during GI were observed among young male adults with normal weight and excess weight in this study. Specifically, we observed that compared with normal-weight young male adults, young male adults with excess weight, mainly those who were obese, exhibited increased CoP path length and higher ML CoP displacement during the sub-phases of GI, while overweight ones did not exhibit differences with normal-weight ones. The results of increased CoP path length suggest that the adductor hip muscles of obese young male adults could have more activations during GI because adductor hip muscles are fundamental for GI in assisting the shifting of CoP from one side leg to the opposite leg for obese young male adults who have poor intrinsic coordination and greater burden in CoP transfer. Once the activation of the hip adductor muscles is reduced, it will result in a shorter CoP path length, as reported by Vrieling et al. (2008) and Cimolin et al. (2017).

However, the increased CoP path length in this study also suggests that obese young male adults could have an unbalance in muscle strength between the adductor and abductor muscles, influencing their postural control in ML direction during GI. The reason for this finding is that dynamic postural balance control needs appropriate intensity muscle contractions of all proximal muscles of the lower limbs during walking, but obese individuals have a relative weakness in the specific contractile performance and quality of hip abductor muscles (Fenato et al., 2021), like gluteus medius, compared with normal-weight ones. The partial lower extremity muscle over-contraction destabilizes the overall body frame and increases the challenge of maintaining postural control in the frontal plane for obese individuals. This finding is consistent with Horsak et al. (2019), the disequilibrium in the strength of the hip abductor and adductor muscles during GI intensifies the level of difficulty in maintaining postural control in the frontal plane for obese individuals. Besides, we also found that obese young male adults have increased displacement amplitude in ML direction during sub-phases of GI, which is consistent with the previous studies that obese individuals have more ML displacement during walking and dynamic balance tests (do Nascimento et al., 2017; McGraw et al., 2000). This finding further proves that, compared with their normal-weight counterparts, obese young males have more difficulty in terms of maintaining lateral stability.

Apart from the CoP parameters, this study showed that although the mean value of the time spent in the imbalance phase of young male adults with excess weight is slightly higher than that of the normal-weight group, there were no significant differences, suggesting regardless of whether young male adults have excess weight or not, the time spent adjusting their posture in advance for the CoP transfer from swing their leg to the stance leg maybe exist slight differences but not obvious enough. Besides, our results suggest that obese young male adults have a prolonged duration of the bipedal standing phase, which may be because more time consumption during this phase is helpful in improving their walking stability, as reported by Duffell et al. (2014). But in contrast with our results, the previous studies believed that obese individuals prolong the imbalance phase to achieve as much posture preparation as possible and take functional adaptation aimed at improving stability by prolonged monopedal standing phase (Cau et al., 2014; Cimolin et al., 2017). The reason for such difference can be attributed to the severity of obesity among the participants in the previous study being much higher than that of the young male adults recruited in our study. Considering that severely obese individuals had the lowest levels of mobility (Hergenroeder et al., 2011), we speculate that this factor amplified the differences in the duration of sub-phases between normal-weight and obese individuals, making their results different from ours. Our findings on the differences in sub-phase duration of GI likely apply more to moderately obese young male adults than severely obese ones.

### 4.2 Cognitive task influences on CoP parameters of young male adults with excess weight

Our results demonstrated that cognitive tasks placed additional demands on cognitive resources, resulting in differences in CoP parameters during GI among young male adults with excess weight and those with normal weight, which supports our assumption 2.

We observed that during the imbalance phase and unloading phase with cognitive tasks, obese young male adults showed distinctly increased ML CoP displacement amplitude. The reason for such results might due to although when encountering cognitive tasks during motion processing, individuals will employ an adaptive postural control strategy to regulate the CoP displacement amplitude during GI to counteract trunk sway with minimal interference from the additional cognitive task (Mille et al., 2014), but most sub-phases of GI put high demands on postural control and once obese individuals face dual-task constraints, their lateral movements and force organization strategy will be modified (Menegoni et al., 2009; Hung et al., 2013; Cimolin et al., 2017), which can lead to abnormal lateral displacement. These findings suggest that for obese young male adults, the compensatory strategy they used for lateral balance during GI with cognitive tasks might have been ineffective, causing deterioration of their postural control and stability.

Moreover, we did not observe that normal-weight and overweight young male adults have significant differences in the parameters of CoP during GI with cognitive tasks. This finding is consistent with Qu et al. (2021), who used random number generation as the cognitive task for participants and did not find differences in the parameters that can reflect postural stability during GI, like margin of stability between normal-weight and overweight young adults. Although they issue that only when the cognitive task reaches a certain level of difficulty, it could amplify differences in postural control status between young male adults with excess weight and normal weight during GI, the cognitive task we employed in this study were N-back tasks which can impose greater cognitive demands than random number generation. Considering this point, we believe that the lack of differences in CoP parameters between normal weight and overweight young male adults during GI with cognitive tasks is not due to insufficient difficulty in cognitive tasks. Instead, it may be because the performance of these young male adults during GI with cognitive tasks may not have obvious differences.

### 4.3 Higher cognitive load influences on CoP parameters of young male adults with excess weight

Consistent with the previous studies that individuals with greater adiposity commonly have poor postural control performance, especially reflected in less effective control in the ML CoP displacement (Tsiros et al., 2019; Saraiva et al., 2023), we observed that obese young male adults showed higher ML CoP displacement and increased CoP path length during GI with difficult-level cognitive tasks, and these results reveal that a higher cognitive load could make it difficult for obese young male adults to maintain postural stability.

It is particularly prioritized for individuals to maintain the magnitude of ML CoP displacements within a reasonable range during GI under dual-task conditions (Uemura et al., 2012; Russo and Vannozzi, 2021). But obese individuals usually need more attentional resources to control their stance leg to maintain postural stability than non-obese participants when performing complex postural control tasks (Mignardot et al., 2010), particularly from the imbalance phase to the unloading phase, individuals need to gradually transfer their entire body weight to the initial stance leg for helping the swing leg step forward (Crenna et al., 2006), higher demand is placed on the ability of obese individuals to maintain balance in supporting themselves on a single-leg compared to single task condition. Difficult cognitive tasks occupied a large number of attentional resources, making obese young male adults’ defects in single-leg control amplified, which could be the main reason for the differences in their displacement amplitude and CoP path length compared to normal-weight young male adults.

Moreover, in this study, we observed that the velocity of AP CoP displacement during the bipedal standing phase with the difficult cognitive task was significantly decreased in obese young male adults compared with the normal-weight ones but not with the overweight ones. This result is inconsistent with our assumption 3. Still, it shows that increased cognitive load during GI will make obese young male adults have obvious differences in CoP parameters compared to normal-weight ones. This finding suggests that the appearance of cognitive tasks has further reduced obese ones’ quality of GI performance due to the velocity of CoP displacement, which is a functional performance indicator, and its value positively correlates with GI performance quality (Esfandiari et al., 2020). The decreased velocity can be attributed to individuals’ cognitive function not being able to meet the requirements of GI and deal with cognitive tasks simultaneously. As reported by Shaik et al. (2022), obesity is typically associated with an increase in young adults’ attentional cost of walking, resulting in a decreased gait speed of dual-task walking.

This result also suggests that when obese young male adults simultaneously dealing with difficult cognitive tasks while GI, they perform more CoP velocity adjustments in the AP direction than their normal-weight counterparts. The reason for such change is that the largest CoP displacement usually occurs during the bipedal standing phase (van Mierlo et al., 2021), and high requirements were placed for individuals to control CoP in the AP direction. But increased attentional demands are required to achieve the cognitive task goal and maintain postural control simultaneously, making excess weight individuals usually produce AP instability in postural control (Menegoni et al., 2009; Hung et al., 2013). The decreased CoP velocity is helping them compensate for a declined ability to perform multiple tasks at once successfully and safely, as reported by the previous studies (Uemura et al., 2012; Cau et al., 2014), that is, accelerating the body forward is not a priority for obese ones, and the reduced velocity in the AP direction is the continuous effort counteracting their relative instability during GI.

### 4.4 Practical implication

This study provided insights into the influences of cognitive load on obese young male adults during GI under dual-task conditions, which could be valuable in the training and rehabilitation of these populations. Firstly, since obese young male adults are unable to correctly move their CoP and transfer body weight as healthy normal-weight do during GI, treatment personnel should modify their dietary habits to improve their metabolic profile and provide them with sufficient exercise to achieve weight loss (Lambert et al., 2017; Balasekaran et al., 2023), which is equally necessary for overweight young male adults. Although the difference between their CoP parameters and normal-weight ones is not significant enough, if their dietary structure is not adjusted, they are also likely to be obese and have similar problems during GI. Secondly, dealing with cognitive tasks can exacerbate lateral posture control disorders during GI. Treatment personnel should pay attention to correcting the unhealthy lifestyle habits of obese ones, such as using their phones while GI (Shahidian et al., 2022). Finally, since obese young male adults have the most pronounced lateral posture control disorders during GI with a higher cognitive load, treatment personnel should provide additional training on the cognitive capacity of obese young male adults to enhance their capacity to make appropriate judgments in complex environments might be greatly beneficial (Shaik et al., 2022), especially effective in reducing lateral falls and preventing related injuries, like femoral neck and hip fractures (Cameron et al., 2011; Wu et al., 2016; Gonzalez et al., 2023).

### 4.5 Limitations

The limitations of this study should be taken into consideration. Following previous research that investigated GI performance in the obese population (Cau et al., 2014; Hirjaková et al., 2018; Qu et al., 2021), this study only grouped young male adults based on BMI and did not use the differentiation between limb and trunk circumference as inclusions for filtering young male adults with excess weight. In addition, although CoP parameters can reflect postural control problems, this study did not analyze kinematic parameters or muscle activation status. Further analysis is needed to examine the differences in these indicators among young male adults with normal weight and excess weight during GI. Moreover, based on the purpose of our study, we only recruited young male adults. Due to the movement performance of individuals with excess weight being different in individuals of different sexes (Menegoni et al., 2009), it is still necessary to conduct similar experiments in young female adults to find their characteristics of CoP parameters during GI. Finally, the number of participants in this study is relatively limited, which may have restricted the efficiency of our results, and there is a need to expand the sample size in future research to verify the findings of this study further.

## 5 Conclusion

The present study preliminarily explores the influence of excess weight and cognitive load on CoP parameters during GI, providing insights into postural control deficiency in young male adults with excess weight. Overall, cognitive load compromises the postural stability of obese young male adults during GI. Especially dealing with higher cognitive load during GI, obese young male adults will experience increased difficulty transferring their CoP, suffer more postural instability and have a higher risk of lateral falling than normal-weight young male adults. Further research will be necessary to determine the most effective exercise or training method for obese young male adults to achieve safe GI.

## Data Availability

The raw data supporting the conclusion of this article will be made available by the authors, without undue reservation.
