# Corrigendum: Influences of cognitive load on center of pressure trajectory of young male adults with excess weight during gait initiation

**DOI:** 10.3389/fbioe.2024.1366726

**Published:** 2024-02-01

**Authors:** Lingyu Kong, Zhiqi Zhang, Jiawei Bao, Xinrui Zhu, Yong Tan, Xihao Xia, Qiuxia Zhang, Yuefeng Hao

**Affiliations:** ^1^ School of Physical Education, Soochow University, Suzhou, China; ^2^ School of Mathematical Sciences, Soochow University, Suzhou, China; ^3^ Rehabilitation Medicine Department, Xuzhou Rehabilitation Hospital, Xuzhou, China; ^4^ Wuxi 9th People’s Hospital Affiliated to Soochow University, Wuxi, China; ^5^ Orthopedics and Sports Medicine Center, The Affiliated Suzhou Hospital of Nanjing Medical University, Suzhou, China; ^6^ Gusu School, Nanjing Medical University, Suzhou, China

**Keywords:** cognition, gait initiation, overweight, obesity, postural control

In the published article, there was an error in the legend for Table 1 as published. The numerical value of an indicator “Preferred swing leg during GI,” which is only used to describe the characteristics of the participants and was not used in statistical analysis, was incorrectly input. The corrected Table 1 appears below.

**TABLE 1:** General characteristics of participants.

Characteristics	Body weight			*F*-value	*p*-value
	Normal-weight (*n* = 12)	Overweight (*n* = 12)	Obese (*n* = 12)		
Age (years)	22.8 ± 2.7	22.9 ± 3.0	22.4 ± 2.5	0.113	0.894
Height (m)	1.72 ± 0.06	1.71 ± 0.04	1.73 ± 0.07	0.514	0.603
Weight (kg)	62.1 ± 7.7	74.5 ± 5.7	93.2 ± 8.1	56.246	**<0.001** ^a^
BMI (kg/m^2^)	20.9 ± 1.5	25.6 ± 1.5	31.1 ± 1.4	152.577	**<0.001** ^a^
Preferred swing leg during GI					
Left	3	7	5	—	
Right	9	5	7		

^a^ *p*-values < 0.017 for normal-weight group vs. overweight group vs. obese group.

The meaning of the bold values is that significant statistical differences exist.

The authors apologize for this error and state that this does not change the scientific conclusions of the article in any way. The original article has been updated.

