# Supplementary material for: Influences of cognitive load on center of pressure trajectory of young male adults with excess weight during gait initiation
Source: Front Bioeng Biotechnol. 2024 Jan 5;11:1297068. doi: 10.3389/fbioe.2023.1297068 (PMC10796550; doi:10.3389/fbioe.2023.1297068)
Supplement: Supplementary file 1 [file DataSheet1.DOCX]

**The** $\boldsymbol{\lambda}$ **values, mean, and 95% confidence intervals** **of the variables transformed using Box-Cox.**

| **Variables** | $\boldsymbol{\lambda}$ | **Mean and 95% confidence interval** | | |
| --- | --- | --- | --- | --- |
|  |  | **Normal-weight** | **Overweight** | **Obese** |
|  |  |  |  |  |
| **Duration** | | | | |
| Imbalance phase | 0.222 | 0.20 (0.10, 0.37) | 0.23 (0.16, 0.31) | 0.23 (0.13, 0.39) |
|  |  | 0.23 (0.14, 0.36) | 0.27 (0.17, 0.42) | 0.27 (0.16, 0.42) |
|  |  | 0.24 (0.15, 0.37) | 0.31 (0.19, 0.47) | 0.30 (0.19, 0.47) |
| Unloading phase | -1.071 | 0.28 (0.15, 3.16) | 0.28 (0.17, 0.89) | 0.27 (0.19, 0.47) |
|  |  | 0.28 (0.19, 0.54) | 0.26 (0.19, 0.39) | 0.29 (0.22, 0.43) |
|  |  | 0.29 (0.20, 0.50) | 0.27 (0.19, 0.46) | 0.27 (0.19, 0.52) |
| **CoP path length** | | | | |
| Unloading phase | 0.101 | 16.55 (8.81, 29.94) | 17.98 (11.17, 28.31) | 18.00 (13.16, 24.38) |
|  |  | 15.04 (9.04, 24.41) | 16.44 (10.44, 25.36) | 19.43 (14.18, 26.37) |
|  |  | 15.63 (10.42, 23.07) | 16.81 (10.27, 26.89) | 21.03 (16.28, 27.00) |
| Monopedal standing phase | 1.838 | 12.27 (2.26, 17.67) | 13.03 (7.20, 17.18) | 13.89 (0.00, 20.40) |
|  |  | 13.27 (6.35, 17.95) | 12.40 (7.93, 15.80) | 12.14 (5.04, 16.72) |
|  |  | 12.43 (2.14, 17.93) | 12.92 (3.14, 18.45) | 12.57 (3.93, 17.73) |
| Bipedal standing phase | 2.000 | 35.59 (19.87, 46.24) | 41.88 (35.74, 47.23) | 44.34 (30.22, 54.94) |
|  |  | 35.93 (19.97, 46.72) | 40.32 (29.79, 48.62) | 43.36 (34.14, 50.94) |
|  |  | 36.98 (26.02, 45.36) | 40.71 (31.35, 48.28) | 39.89 (30.11, 47.70) |
| **CoP speed** | | | | |
| Imbalance phase | 0.384 | 27.56 (4.47, 79.63) | 29.30 (9.39, 64.50) | 30.48 (8.16, 72.80) |
|  |  | 22.25 (7.24, 48.66) | 26.82 (15.30, 42.55) | 31.41 (10.21, 68.69) |
|  |  | 25.57 (14.03, 41.63) | 24.67 (12.06, 43.23) | 30.44 (15.12, 52.82) |
| Unloading phase | 0.222 | 55.09 (11.46, 176.04) | 60.82 (22.19, 138.54) | 66.50 (35.71, 114.82) |
|  |  | 53.28 (25.84, 99.36) | 63.29 (38.88, 98.24) | 67.06 (42.47, 101.51) |
|  |  | 53.36 (31.17, 86.24) | 61.92 (34.52, 103.86) | 75.59 (47.35, 115.48) |
| Bipedal standing phase | -0.263 | 191.01 (133.45, 283.78) | 210.46 (159.38, 284.05) | 211.27 (134.20, 353.70) |
|  |  | 186.06 (122.11, 298.77) | 191.67 (137.56, 275.67) | 178.03 (117.82, 282.87) |
|  |  | 190.88 (131.35, 288.91) | 192.13 (133.33, 287.81) | 155.16 (110.46, 225.34) |

| **Variables** | $\boldsymbol{\lambda}$ | **Mean and 95% confidence interval** | | |
| --- | --- | --- | --- | --- |
|  |  | **Normal-weight** | **Overweight** | **Obese** |
|  |  |  |  |  |
| **CoP displacement amplitude** | | | | |
| Unloading phase AP | 0.545 | 2.51 (0.35, 6.35) | 2.67 (0.42, 6.60) | 1.57 (0.03, 5.00) |
|  |  | 2.75 (0.43, 6.78) | 1.51 (0.07, 4.47) | 1.18 (0.12, 3.16) |
|  |  | 2.36 (0.00, 9.48) | 1.48 (0.01, 4.97) | 1.89 (0.04, 6.06) |
| Monopedal standing phase AP | 1.879 | 11.15 (0.67, 16.11) | 12.07 (6.39, 16.00) | 12.55 (0.00, 18.22) |
|  |  | 12.19 (5.39, 16.59) | 11.34 (6.67, 14.72) | 10.98 (5.09, 14.86) |
|  |  | 11.58 (0.00, 16.99) | 11.98 (1.52, 17.23) | 11.17 (4.46, 15.37) |
| Monopedal standing phase ML | 0.667 | 2.25 (0.00, 6.32) | 2.43 (0.34, 5.55) | 3.14 (0.71, 6.53) |
|  |  | 1.82 (0.00, 5.60) | 2.53 (0.32, 5.84) | 2.66 (0.13, 6.80) |
|  |  | 1.81 (0.00, 5.25) | 2.03 (0.08, 5.26) | 2.96 (0.71, 6.06) |
| Bipedal standing phase AP | 1.960 | 30.82 (18.16, 39.74) | 34.88 (24.50, 42.89) | 34.57 (22.30, 43.61) |
|  |  | 30.68 (16.77, 40.15) | 33.47 (22.87, 41.53) | 32.92 (27.91, 37.28) |
|  |  | 31.6 (20.03, 40.04) | 32.51 (21.74, 40.60) | 29.71 (22.96, 35.23) |
| **Velocity of CoP displacement** | | | | |
| Imbalance phase AP | 0.505 | 13.74 (0.61, 43.66) | 14.76 (3.76, 32.90) | 15.44 (1.47, 43.90) |
|  |  | 13.90 (3.18, 32.06) | 14.67 (7.01, 25.09) | 14.60 (2.95, 34.93) |
|  |  | 12.72 (2.86, 29.52) | 13.77 (5.46, 25.81) | 13.69 (2.10, 35.26) |
| Imbalance phase ML | 0.505 | 22.01 (2.04, 62.88) | 23.91 (6.09, 53.30) | 23.49 (5.46, 53.92) |
|  |  | 15.13 (2.64, 37.71) | 21.27 (11.80, 33.48) | 26.28 (7.27, 56.91) |
|  |  | 16.95 (5.21, 35.32) | 18.74 (8.94, 32.06) | 24.38 (10.00, 45.00) |
| Unloading phase AP | 0.424 | 8.59 (0.29, 32.63) | 8.68 (1.42, 23.84) | 5.33 (0.41, 17.70) |
|  |  | 9.21 (2.76, 20.39) | 5.59 (0.35, 19.25) | 4.02 (0.42, 12.43) |
|  |  | 7.44 (0.00, 38.26) | 5.16 (0.08, 21.45) | 6.70 (0.06, 29.08) |
| Unloading phase ML | 0.384 | 50.70 (6.05, 160.89) | 56.01 (13.97, 137.93) | 63.55 (31.24, 110.98) |
|  |  | 47.18 (18.20, 94.63) | 60.12 (35.49, 93.19) | 64.33 (38.91, 98.03) |
|  |  | 46.74 (23.81, 79.84) | 59.43 (31.16, 99.66) | 72.27 (43.94, 109.74) |
| Monopedal standing phase ML | 0.545 | 8.27 (0.01, 28.79) | 7.91 (1.16, 19.79) | 10.33 (2.27, 23.43) |
|  |  | 6.93 (0.08, 22.74) | 8.04 (1.55, 18.86) | 9.01 (0.52, 26.20) |
|  |  | 6.97 (0.15, 22.13) | 6.77 (0.07, 22.40) | 9.70 (2.53, 20.91) |
| Bipedal standing phase AP | -0.222 | 165.39 (110.95, 256.31) | 173.20 (117.78, 264.07) | 164.41 (100.91, 284.29) |
|  |  | 158.49 (104.47, 250.89) | 158.31 (112.82, 228.36) | 136.03 (94.71, 201.69) |
|  |  | 161.62 (107.23, 253.83) | 151.90 (102.04, 235.02) | 115.89 (82.93, 166.36) |
| Bipedal standing phase ML | 0.101 | 89.16 (56.82, 137.18) | 108.26 (70.41, 163.50) | 126.93 (73.58, 212.83) |
|  |  | 90.85 (46.29, 170.83) | 98.81 (61.69, 154.92) | 108.70 (56.27, 201.55) |
|  |  | 93.15 (55.55, 152.25) | 101.82 (57.75, 174.08) | 105.70 (66.41, 164.75) |
